# A scoping review of machine learning for sepsis prediction- feature engineering strategies and model performance: a step towards explainability

**DOI:** 10.1186/s13054-024-04948-6

**Published:** 2024-05-28

**Authors:** Sherali Bomrah, Mohy Uddin, Umashankar Upadhyay, Matthieu Komorowski, Jyoti Priya, Eshita Dhar, Shih-Chang Hsu, Shabbir Syed-Abdul

**Affiliations:** 1 Graduate Institute of Biomedical Informatics, College of Medical Science and Technology, Taipei Medical University, No. 291, Zhongzheng Rd, Zhonghe District, New Taipei City, 235 Taiwan; 2 International Center for Health Information Technology, College of Medical Science and Technology, Taipei Medical University, Taipei, 235 Taiwan; 3 College of Medicine, Taipei Medical University, Taipei, 110 Taiwan; 4 Research Quality Management Section, King Abdullah International Medical Research Center, King Saud Bin Abdulaziz University for Health Sciences, Ministry of National Guard—Health Affairs, 11426 Riyadh, Saudi Arabia; 5 School of Biotechnology and Applied Sciences, Shoolini University of Biotechnology and Management Sciences, Solan, 173229 India; 6 Faculty of Medicine, Department of Surgery and Cancer, Imperial College of London, South Kensington Campus, London, UK; 7 Department of Emergency, School of Medicine, College of Medicine, Taipei Medical University, Taipei, 106 Taiwan; 8 Emergency Department, Wan Fang Hospital, Taipei Medical University, Taipei, 116 Taiwan; 9 School of Gerontology and Long-Term Care, College of Nursing, Taipei Medical University, Taipei, Taiwan

**Keywords:** Machine learning, Sepsis prediction, Scoping review, Critical features, Performance evaluation, Clinical outcome, Feature engineering

## Abstract

**Background:**

Sepsis, an acute and potentially fatal systemic response to infection, significantly impacts global health by affecting millions annually. Prompt identification of sepsis is vital, as treatment delays lead to increased fatalities through progressive organ dysfunction. While recent studies have delved into leveraging Machine Learning (ML) for predicting sepsis, focusing on aspects such as prognosis, diagnosis, and clinical application, there remains a notable deficiency in the discourse regarding feature engineering. Specifically, the role of feature selection and extraction in enhancing model accuracy has been underexplored.

**Objectives:**

This scoping review aims to fulfill two primary objectives: To identify pivotal features for predicting sepsis across a variety of ML models, providing valuable insights for future model development, and To assess model efficacy through performance metrics including AUROC, sensitivity, and specificity.

**Results:**

The analysis included 29 studies across diverse clinical settings such as Intensive Care Units (ICU), Emergency Departments, and others, encompassing 1,147,202 patients. The review highlighted the diversity in prediction strategies and timeframes. It was found that feature extraction techniques notably outperformed others in terms of sensitivity and AUROC values, thus indicating their critical role in improving sepsis prediction models.

**Conclusion:**

Key dynamic indicators, including vital signs and critical laboratory values, are instrumental in the early detection of sepsis. Applying feature selection methods significantly boosts model precision, with models like Random Forest and XG Boost showing promising results. Furthermore, Deep Learning models (DL) reveal unique insights, spotlighting the pivotal role of feature engineering in sepsis prediction, which could greatly benefit clinical practice.

## Introduction

Sepsis, a severe and life-threatening condition triggered by an overwhelming immune response to infection, poses a significant global health challenge [1]. It is responsible for an estimated 31.5 million cases of sepsis and 19.4 million cases of severe sepsis annually, leading to approximately 5.3 million deaths worldwide [2]. The critical nature of timely sepsis identification in clinical practice is underscored by findings that even a brief delay in initiating treatment can substantially increase mortality rates, owing to irreversible organ damage[3]. This urgency has catalyzed research into advanced predictive methodologies, notably the application of Machine Learning (ML) techniques aimed at the early detection of sepsis[4]. Such research endeavors have largely concentrated on the development of ML models and tools with a focus on improving prognosis, diagnosis, and the integration of clinical workflows, thereby highlighting the potential for constructing sophisticated computerized decision support systems[5].

Despite these advancements, traditional sepsis prediction methodologies, including the Sequential Organ Failure Assessment (SOFA), Systemic Inflammatory Response Syndrome (SIRS), and quick SOFA (qSOFA), exhibit significant limitations[6]. These methods often rely heavily on clinical judgment and are subject to variability in interpretation across different levels of clinical expertise, which can lead to inconsistencies in early sepsis detection. Moreover, traditional approaches tend to identify sepsis at a more advanced stage, missing the crucial window for early intervention [6, 7]. Conversely, ML models offer a dynamic and continuous analysis of real-time patient data, enabling early detection and providing dynamic risk assessments. By harnessing data analysis and pattern recognition capabilities, ML models aim to enhance patient outcomes and alleviate the burden on healthcare systems[7].

Feature engineering emerges as a critical component in the optimization of ML models for sepsis prediction. This process entails the selection, transformation, and creation of relevant features from raw data, aiming to improve the predictive accuracy of models. While the significance of identifying and utilizing critical features in constructing robust and precise predictive models is well-recognized, the field continues to grapple with uncertainties surrounding the effectiveness of specific features and the methodologies for feature selection and extraction[8]. Variability in the approaches to feature engineering and their impact on model performance necessitates a comprehensive scoping review to evaluate the evidence and discern which strategies yield the most significant benefits in terms of prediction accuracy.

In the context of feature engineering for sepsis prediction, the current literature has focused on the importance of selecting and extracting the most relevant patient-related variables to enhance the model accuracy [9]. Though some studies have explored clinical and laboratory features to improve sepsis prediction models, such as vital signs (e.g., temperature, heart rate, respiration rate, blood pressure), laboratory values (e.g., white blood cell count, lactate levels), patient demographics, and clinical history to improve sepsis prediction models; but still there is uncertainty regarding the effectiveness of specific features and feature selection/extraction methods in sepsis prediction[10]. Our current study employed different approaches to feature engineering, and their impact on model performance varies. This variability highlights the need for a scoping review to comprehensively evaluate the available evidence and provide insights into which feature-engineering strategies offer the greatest benefits in terms of sepsis prediction accuracy.

This scoping review seeks to address these gaps by assessing the critical features that enhance sepsis prediction and by providing insights into identifying patterns that may lead to improved clinical outcomes. The primary objective of this review is twofold: firstly, to explore the feature engineering strategies utilized in ML models for sepsis prediction, thereby offering valuable insights for future research and model development; and secondly, to evaluate the performance of these models through a critical analysis of existing studies, focusing on metrics such as the Area Under the Receiver Operating Characteristic curve (AUROC), Sensitivity, and Specificity.

Through an exhaustive evaluation of 29 selected studies, this review aims to analyze and synthesize various feature engineering techniques applied in sepsis prediction models, assess their impact on model performance, and evaluate the overall effectiveness of ML models in predicting sepsis. By adopting a systematic approach, the review intends to provide a comprehensive understanding of the role of feature engineering in enhancing sepsis prediction models, ultimately contributing to more effective clinical decision-making and patient care.

## Methods

### Search strategy

**Fig. 1:**
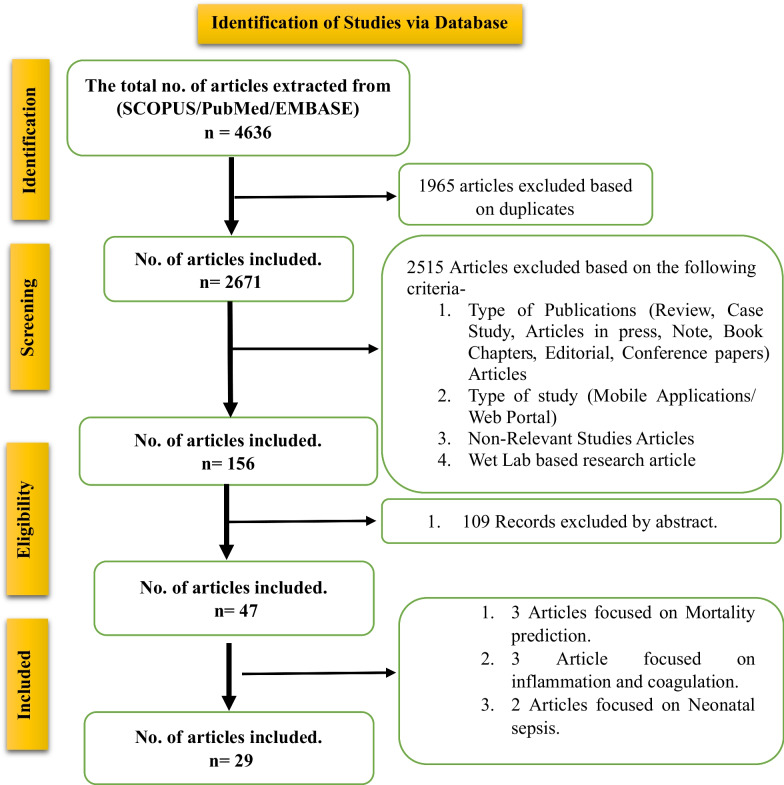
Preferred Reporting Items for Scoping reviews and Meta-Analysis (PRISMA) flow diagram for the conducted study

The search strategy for this scoping review was meticulously devised in alignment with the Preferred Reporting Items for Scoping Reviews (PRISMA) guidelines. PRISMA represents a rigorously developed framework, outlining a comprehensive set of standards for reporting scoping reviews. This methodology ensures transparency and reproducibility in the review process, as illustrated in Figure 1.[11]

On 13th March 2023, a comprehensive literature search was conducted across PubMed, Embase, and Scopus, targeting publications from the past five years (13 March 2018 to 13 March 2023). This search employed a detailed strategy, utilizing Boolean operators "AND" and "OR" to combine key phrases, specifically: "Machine Learning" and "Prediction" along with "Sepsis" and "Septic Shock". Each database was queried with these terms to ensure a thorough retrieval of relevant studies. Subsequently, the titles and abstracts of the retrieved studies were meticulously reviewed by an investigator (SB) to ascertain their suitability for inclusion in the review.

### Inclusion and exclusion criteria

**Inclusion criteria:**

- Research articles published in the English language.
- Studies appearing in peer-reviewed journals.
- Research focusing on the prediction of sepsis and associated outcomes.
- Studies investigating ML (Machine Learning) models for sepsis prediction, emphasizing significant features for model optimization.

**Exclusion criteria:**

- Conference abstracts and preliminary proof of concept studies.
- Research studies exclusively predicting mortality related to sepsis.
- Research studies published in the subscribed journals

These criteria ensured a comprehensive and focused review of the literature on ML models for sepsis prediction by excluding preliminary findings and studies that were not directly aligned with the core objectives of efficient prediction and feature analysis.

### Data extraction and quality assessment

Data extraction was meticulously carried out by a primary reviewers (SB) and (JP), who cataloged essential details such as Title, Publication year, First author, Study objectives, Clinical setting, Patient cohort size, ML model utilized, Feature count, Sepsis classification, Observation period, Gender distribution, AUROC, Innovation, Model evaluation criteria,Training-test split, Data source, Sensitivity, and Specificity, in addition to the criteria used for sepsis diagnosis.

To ensure the accuracy and integrity of the data extraction process, two additional reviewers (ED and UU) collaboratively worked with the primary reviewer (SB) to scrutinize and validate the extracted information. Studies failing to align with the predetermined inclusion criteria were systematically excluded. Discrepancies encountered during the review process were resolved through comprehensive mutual discussion and further literature consultation, facilitating consensus among the reviewers.

## Results

### Characteristics of studies

The scoping review included 29 studies (See in Table 1), encompassing a total patient cohort of 1,147,202 (909,462 cases and 237,740 controls). The majority of the studies, numbering 20, (3,4,12,13,17,18,19,20,21,5,23,24,25,28,30,31,32,33,34,36) were conducted in Intensive Care Units (ICUs), while four were based in Emergency Departments (EDs) 16,26,29,35, and one was carried out in a general hospital setting[14]. These studies varied in patient demographics, prediction timeframes, and sepsis types, utilizing diverse database sources. For instance, the research by Meicheng Yang et al. [3]focused on hourly sepsis risk prediction in ICU settings with the EASP model, emphasizing interpretability. Maximiliano Mollura et al. [12] and Xin Zhao et al. [13] explored ICU data and PhysioNet/Clinic Challenge 2019 data, respectively, each applying distinct approaches to sepsis prediction and addressing specific challenges.

**Table 1:** Characteristics of included studies

References	Setting	No. of Patients	Prediction Window	Types of Sepsis	Database Source	Novelty
Meicheng Yang et al. [3]	ICU	Sepsis:2,932 Non-Sepsis:37,404	6 h	Sepsis 3	PhysioNet/Clinic Challenge 2019	EASP predicts sepsis risk hourly, emphasizing interpretability
Maximiliano Mollura et al. [12]	ICU	10,282	48 h	Sepsis 3	MIMIC-III	Framework extracts parameters, characterizes patient states, explains sepsis
Xin Zhao et al. [13]	ICU	Sepsis:20,662 Non Sepsis:1,714	6,12,24 h	Sepsis 3	PhysioNet/Clinic Challenge 2019	Rules predict early sepsis, data interference avoided
Debdipto Mishra et al. [14]	ICU	Sepsis:5,784 Control:30,192	1,3,6 h	Sepsis Infection + SIRS	EPIC, AMISYS	Direct ML based system for septic shock detection
Donghun Yang et al. [15]	ICU & ER	Sepsis:455 Control:928	2 to 7 days	Sepsis 3	Clinical Data Warehouse (CDW) and the SMC Cancer Registry, Seoul, South Korea	Efficient sepsis prediction in cancer patients
Gabriel Wardi et al. [16]	ED	Case:8,499 Control:6,409	8,12,16,24,36 h	Sepsis 3	MIMIC-III	Transfer learning improves septic shock prediction
Ekanath Srihari Rangan et al. [17]	ICU	Case:1,130 Control:1,500	3,4,5,6 h	Sepsis 3	Telehealth Intensive Care Unit and MIMIC-IV	Body temperature amplifies sepsis prediction (AUC-0.9)
Yu Bai et al. [18]	E-ICU	Case:5,947 Control:13,302	24 h	ICD-9-CM, ICD-10-CM,	MIMIC-IV	Unexplored ML in sepsis-associated ARDS prediction and classification
Zhengling He et al. [19]	ICU	40,336	6 h	SIRS + SOFA	PhysioNet/Computing in Cardiology Challenge 2019	ICU length of stay crucial in FRAW; LSTM features effective
Brandon DeShon et al. [1]	ICU,TCU, ED	Case:2,919 Control:32,095	21 h	SIRS + Sepsis 3	EMR DATABASE	Survival analysis predicts sepsis and shock using diverse data
Everton Osnei Cesario et al. [20]	Infirmary & ICU	Case:4,331 Control:479	1,3,5,8 days	ICD10 + qSOFA	Brazilian Hospital	Age emerges as pivotal feature for sepsis prediction
Kim Huat Goh et al. [21]	ICU	3722 (240-Sepsis Patient in Training and validation sample) and (87 Sepsis patient in Test Sample)	4,6,12,24,48 h	ICD-10	Singapore Govt based Hospital	NLP uncovers valuable insights from clinician progress notes
Dong Wang et al. [5]	ICU	Case:3,539 Control:910	NR	Sepsis 3	Affiliated hospital of ZHENGZHOU University	Neglected electrolyte-sepsis link: potassium and magnesium impact
Jevier Enrique Camacho- Cogollo et al. [22]	ICU	Case:537 Control:1,840	24,12,6,1 h	Sepsis 3	MIMIC-III	Enhanced sepsis prediction: ensemble models and innovative feature selection
Bilal Yaseen Al-Mualemi et al. [23]	ICU	40,336	4 h	SIRS	Not Mentioned	Exploring clinician perspectives on early sepsis detection tools
Margherita Rosnati et al. [24]	ICU	Case:7,936 Control:14,071	0 to 6 h	Sepsis 3	MIMIC-III	Enhanced sepsis detection using MGP-attTCN: a novel ML approach
Rishikesan Kamaleswaran et al. [25]	ICU	(Sepsis Non-Transplant) 604 Case,5144 Control Sepsis Transplant 52 Case, 160 Control	up to 24 h	Sepsis 3	ICU Unit from methodist University Hospital and Transplant Institute	Utilizing bedside monitoring data for CDS in Post-liver transplant patients
Pei Chen Lin et al. [26]	ED	Case:8,296 Control:1,744	3.18 h	Sepsis 3	Chi-Mei Medical Centre and the Taoyuan General Hospital	External validation of ML for sepsis identification outperforms SIRS and qSOFA criteria
Supreeth P. Shashikumar et al. [27]	ICU & ED	515,720	4 to 48 h	Sepsis 3	University of California San Diego Health and Emory University hospital in US	Conformal prediction framework for clinical implementation of sepsis prediction algorithms
Jacob Calvert et al. [28]	ICU	122,672	3 h	ICD-9 + Organ Dysfuntion	MIMIC-III, Stanford Medical Centre in Stanford, San Francisco Medical Centre	ML diagnosis outperforms traditional criteria and biomarkers for sepsis diagnosis
Massimiliano Greco et al. [29]	ED	425	NR	ICD-9	Tertiary Clinical Centre	RF model outperforms traditional scoring systems in identifying high mortality risk sepsis patients
Kuo- Ching Yuan et al. [30]	ICU	Sepsis:444 Non-Sepsis:1,144	1 to 6, 7 to 12 h	Sepsis 3	TMU Hospital	XG boost algorithm outperforms SOFA score in early sepsis detection
Yash Veer Singh et al. [31]	ICU	1,572	NR	Sepsis 2,3	Skaborg Hospital	Proposed method enhances classification performance
Jae Kwan Kim et al. [32]	ICU	Case:27,670 Control:27,670	0 to 12 h, 3 h	ICD-9	MIMIC-III	Automated model generates adaptive neural network architectures for enhanced prediction
Yongrui Duan et al. [33]	ICU	282	6,12,24 h	Sepsis2	Shanghai Hospital	Integrating deep learning and clinical knowledge for improved predictive modeling in clinical settings
Simon Meyer Lauritsen et al. [34]	ICU	3,126	3 h,24 h,10 h	ICD-10	CROSS-TRACKS	Novel technique to assess model utility incorporating antibiotics & blood culture requisition
Heather M. Giannini et al. [35]	Non-ICU	Silent: 22,280 Alert: 32,184	24 h	Sepsis 3	University of Pennsylvania	Feasibility of real-time EHR-based ML for accurate sepsis prediction
Matthieu Scherpf et al. [36]	ICU	Case:6,688 Control:30,754	3,6,12 h	SIRS + Infection	MIMIC-III	Valuable incremental information in sepsis development
Alireza Rafiei et al. [4]	ICU	40,336	4,8,12 h	Sepsis 3	2019 PhysioNet/ Computing in Cardiology Challenge database	SSP: Predicting sepsis up to 12 h in advance

^**^ED: Emergency Department, E-ICU: Electronic Intensive Care Unit, ER: Emergency Room, EMR: Electronic Medical Record, ICU: Intensive Care Unit, MIMIC: Medical Information Mart for Intensive Care, SSP: Smart Sepsis Predictor, TCU: Transitional Care Unit, TMU: Taipei Medical University, NR: Not Reported

**Fig. 2:**
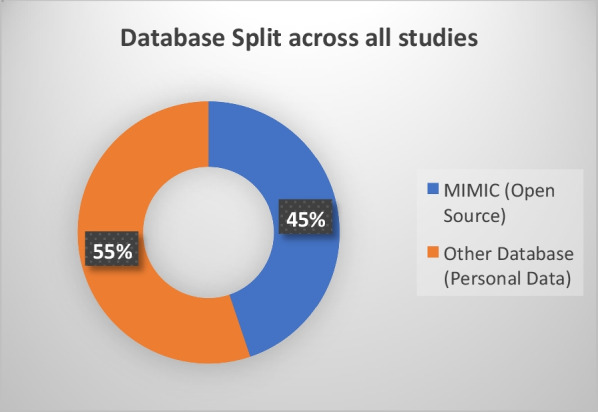
Database sources used in the studies

Figure 2's pie chart illustrates the data source distribution, revealing a nearly equal split between private (55%) and public databases (45%), such as MIMIC, indicating the varied origins of data in these studies. This comprehensive review underscored the array of strategies and methodologies employed to enhance sepsis prediction across different clinical environments, contributing to the ongoing advancement in the field.

### Feature engineering techniques

**Fig. 3:**
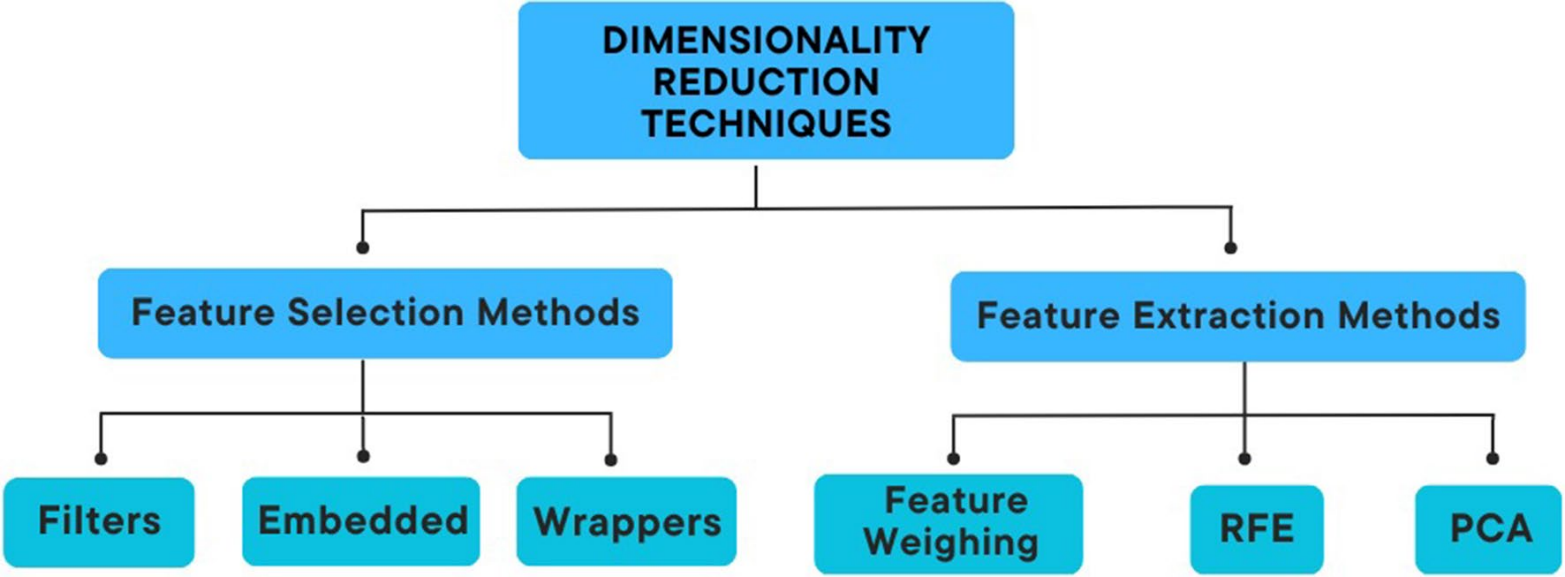
Classification of feature selection methods

Feature selection is a process of selecting feature subsets which are applied to the model construction. It is used in areas where there any many features and relatively few samples. On the other hand, feature extraction generates new features from the original features, which means that the new features after feature extraction is a mapping of the original features (See Figure 3). [35]

#### Feature selection methods: Filter methods

Filter methods employ variable ranking techniques as their core criterion for feature selection, arranging variables based on their relevance. This relevance, termed feature relevance, measures a feature's utility in distinguishing between different classes within the data. Utilizing methods like Info Gain, GINI, and Relief, Jevier Enrique Camacho-Cogollo et al.[20] applied the filter approach to score and rank features according to their class label relevance, selecting features above a specified relevance threshold (0.0020). This process identified 31 medically relevant features and 88 statistical features, with Info Gain, GINI, and Relief methods selecting 75, 47, and 76 relevant features respectively. Similarly, Donghun Yang et al. [15] employed the filter method to narrow down from 1,738 initial features to the 50 most critical features, encompassing both laboratory data and drug interactions, thereby underscoring their significance in enhancing the accuracy of their predictive model.

#### Feature selection methods: Wrapper methods

Wrapper methods optimize feature selection by treating the prediction model as a “black box," utilizing the model's performance metrics as the objective function for evaluating subsets of variables. [2]This approach typically yields higher performance subsets than filter methods by leveraging actual modeling algorithms for evaluation.[38] Yash Veer Singh et al.[31] applied backward elimination, a wrapper method, effectively removing non-contributory features to identify 11 critical features, achieving a model accuracy of 0.96 with their Ensemble model. Meicheng Yang et al. employed forward feature selection, another wrapper strategy, categorizing their 168 selected features into raw features, information missingness, time series, and empiric categories, showcasing the adaptability of wrapper methods in refining feature sets for predictive modeling.

#### Feature selection methods: Embedded methods

Embedded methods integrate the feature selection process directly within the training phase of ML models, offering a nuanced approach that inculcates the complexity of model training with the simplicity of feature optimization. These methods, such as Lasso and Elastic Net, operate on the principle of regularization, which aims to minimize overfitting by penalizing the magnitude of feature coefficients, effectively shrinking some to zero. [38] This not only aids in identifying features that have little to no predictive value but also enhances model generalizability.

In the realm of sepsis prediction, embedded methods have shown considerable promise. For instance, the use of Random Forest importance as an embedded method highlights its capability to discern the relative value of each feature within a dataset. By analyzing feature importance, researchers can pinpoint which variables most significantly impact the model's predictions, particularly in the context of sepsis where timely and accurate prediction can save lives. Dong Wang et al.'s [5] application of this method led to the selection of a concise set of 20 features critical for sepsis prediction in ICU patients, underscoring the method's efficiency in distilling a dataset to its most informative components.

Further exploration by Cesario et al. [20]into Mean Decrease Accuracy and Mean Decrease GINI as embedded methods provides insights into the multifaceted nature of feature selection. These techniques evaluate the impact of each feature on the model's accuracy and the overall reduction in data impurity, respectively, offering a comprehensive view of feature significance. Such methodologies have elucidated the paramount importance of certain predictors, like age, which exhibited a profound influence on the model's predictive capabilities.

Rishikesan Kamaleswaran et al.'s [25] study stands out for its broad application of feature selection methods, spanning both embedded and wrapper techniques. By employing a wide array of methods, including parametric and non-parametric tests, Ridge, Lasso, and Recursive Feature Elimination (RFE), alongside Random Forest-based variable importance, the study showcases the depth of possible analysis when integrating feature selection with model development. The adoption of Recursive Feature Elimination, in particular, highlighted its effectiveness in isolating 22 highly predictive features, demonstrating the potential of embedded methods to refine and enhance model performance through targeted feature selection.

This comprehensive approach to feature selection, particularly within the scope of embedded methods, exemplifies the dynamic interplay between algorithmic complexity and model optimization. By embedding feature selection within the model training process, these methods provide a robust framework for developing highly accurate and generalizable predictive models, essential for advancing sepsis prediction and improving patient outcomes.

#### Feature extraction methods

Zhengling He et al. [19] and colleagues explored the potential of LSTM (Long Short-Term Memory networks) for deriving features from sequential data, employing an ablation study to gauge the impact of individual features. Their findings highlight the ICU Length Of Stay (LOS) as a pivotal predictor, alongside other significant LSTM-derived features like the pseudo SOFA score and body temperature. These insights underscore the value of deep learning in identifying nuanced indicators for sepsis onset prediction.

Further innovation in feature engineering was demonstrated through the development of second-order derived features and aggregate features [17], capturing complex relationships and condensing data into insightful metrics. This approach yielded a comprehensive set of 672 features, with 192 identified as unique, revealing the synergistic effect of body temperature and heart rate, among others, on sepsis prediction accuracy and lead time.

Table 2 consolidates various feature selection and extraction methods, ranging from wrapper and embedded methods to unsupervised techniques, highlighting their effectiveness in distilling critical predictors from a broad spectrum of clinical and demographic data. This table illustrates the evolution from initial feature identification to the final selection, emphasizing the top ten features across studies, and showcasing the diversity and impact of feature selection and extraction strategies on enhancing model performance.

**Table 2:** Feature Selection Methods and Important Features in the Included Studies

References	Feature selection/extraction method	Total. no. of Features	No. of Final Features	Top 10 Features
*Intensive care unit*				
Meicheng Yang et al. [3]	Wrappers	168	20	ICULOS, Hospital Admission, Time, Temp, Fio2, Fio2_interval, Lactate, WBC, Creatinine, Unit 1, BUN
Maximiliano Mollura et al. [12]	Embedded + Wrapper	75	30	SDPAT, SD_Ratio, PAT_HF, AVPAT, Vent_Flag, NN50, pNN50, AVSAP, Avg _ssr_hfn, DAP_VLF
Xin Zhao et al. [13]	Embedded	40	25	Temp, O2Stat, Resp, BUN, Magnesium, HR, Potassium, Bilirubin_total, DBP, PTT, PH
Ekanath Srihari Rangan et al. [17]	Feature Extraction (2nd order derived aggregate features)	672	240	HR, Temp (baseline),Respiration, Temp Variance,SP02,HR(baseline), SP02(Delta between 2 and baseline), Temp(between 4 and 3)
Yu Bai et al. [18]	Unsupervised	27	27	APACHE_4,HC03_max,Lactate_Max,Lactate_Min,HC03_Min,Creatinine_Min, Albumin_Min, Creatinine_Max, Albumin_Max and Glucose_Min
Zhengling He et al. [19]	Feature Extraction (LSTM)	82	82	Bilirubin_total, Creatinine, Fi02, HR, MAP, PaCo2, Platelets, RR, SBP, SIRS_Resp
Everton Osnei Cesario et al. [20]	Embedded	16	16	Age, DBP, HR, SBP, RR, Blood Glucose, Admission Days, Temp, Gender, Surgical Procedure (for RF)
Kim Huat Goh et al. [21]	Filter Method	100 Topics	100 Topics	NR
Dong Wang et al. [5]	Embedded	55	20	Neutrophil%, D-Dimer, Neutrophils, Eosinophils %,Lymphocyte %, Albumin,WBC,Direct Bilirubin, Potassium and Calcium
Jevier Enrique Camacho- Cogollo et al. [22]	Filter Method	913	Infogain:75 Gini:47 Relief:76	Min Glasgow Score, Temp_min, Glucose, SP02_max, HR_Min. Meanbp_min, Ph_max, FE2,Temp_max, DiasBP_min
Bilal Yaseen Al-Mualemi et al. [23]	Feature Extraction (ACNN)	34	7	NR
Margherita Rosnati et al. [24]	Embedded	24	24	SBP, DBP, Mean BP, RR, HR, Sp02_pulsary, Temp., Bicarbonate, Creatinine, Chloride
Rishikesan Kamaleswaran et al. [25]	Embedded + Wrapper	311	Ridge:52 Lasso:12 RFE:22	SBP_SD, SBP_sum_values, RR_mean, SBP_mean, SBP_min, SBP_max, HR_length, SBP_median, RR_sum_value, RR_min
Jacob Calvert et al. [28]	Filter Method	6	6	NR
Kuo- Ching Yuan et al. [30]	Feature Extraction & Filter (Feature Weight)	106	5	Infection (any site), Resp_Infect, Neuro_Infect, LAB_CRP, LAB_WBC, UT_Infect, GI_Infect, HBT_Infect, Skin_Infect, CVS_Infect
Yash Veer Singh et al. [31]	Wrapper & Feature Extraction (PCA)	23	23	Age, Gender, Temp, RR, HR, SBP, DBP Positive Blood Culture, MAP, Lactate, WBC
Jae Kwan Kim et al. [32]	Embedded	13,000	40	Age, HR, SBP, Temp, RR, GCS, Mechanical Ventilation, Pa02, Fi02, Urine Output
Yongrui Duan et al. [33]	Feature Extraction (Early Fusion)	451	451	NR
Simon Meyer Lauritsen et al. [34]	Feature Extraction	30	30	NR
Matthieu Scherpf et al. [36]	Feature Extraction	101	101	NR
Alireza Rafiei et al. [4]	Feature Extraction (LSTM)	14	14	NR
*Emergency department*				
Gabriel Wardi et al. [16]	Embedded	40	20	SBP, BUN, RR, Temp, ΔSBP, HCT, WBC, Lactate, Creatinine, HR
Pei Chen Lin et al. [26]	Embedded	15	15	C-Reactive Protein, Sodium level, lymphocyte, Creatinine, Blood Temp, Platelet, Red Cell Distribution Width, GPT, HB, Segment
Massimiliano Greco et al. [29]	Embedded	40	40	Age, Sodium. HR, CRP, Potassium, RR, Neutrophil, p02, SOFA, HC03
Heather M. Giannini et al. [35]	Embedded	587	48 +	NI DBP, Non Invasive SBP, Pulmonary Service, HR, BUN, BP, Temp. (most recent), Temp. (24 h. max), %Monocytes and Temp (24 h. variation)
*In-Hospital*				
Debdipto Mishra et al. [14]	Unsupervised	65	15	Lactic Acid, SVP, Blood Culture, Creatinine, MAP, Whole Blood Count, Platelet Count, Respiration, Pulse, DBP
*Combined*				
Donghun Yang et al. [15] (ICU&ED)	Filter Method	1738	50	Albumin, Platelet Count (Blood), Bilirubin (Total), PT(INR), A/G Ratio, Protein Total Cholesterol, ANC, AST, Calcium
Brandon DeShon et al. [1] (ICU,TCU,ED)	NR	NR	NR	Age, Weight, GCS, Platelets, BUN, Creatinine, Arterial_pH, Temp, RR, WBC Count
Supreeth P. Shashikumar et al. [27] (ICU, ED)	Feature Extraction	108	40	Temp, BUN, Baseline WBC, ΔWBC, ΔTemp, HR, Elapsed Time, RR, Baseline HR, Baseline Platelets

A/G Ratio: Albumin/Globulin Ratio, ALC: Absolute Lymphocyte Count, ALP: Alkaline Phosphatase, ANC: Absolute Neutrophil Count, APACHE: Acute Physiology and Chronic Health Evaluation, AST: Aspartate Aminotransferase, AVPAT: Average of PAT, BUN: Blood Urea Nitrogen, CRP:C-Reactive Protein, DAP_VLF: Diastolic Arterial Pressure Very Low Frequency, DBP: Diastolic Blood pressure, Fio2: Fraction of Inspired Oxygen, GCS: Glasgow Coma scale, GI: Gastrointestinal, GPT: Glutamate pyruvate transaminase, HBT: Hydrogen Breadth Test,HCO3: Bi Carbonate, HCT: Haematocrit Test, HF: High Frequency, HR: Heart Rate, ICULOS: ICU Length of Stay, INR: International Normalized Ratio, MAP: Mean Average Precision, Max: Maximum, Min: Minimum, NN 50: Neural Network 50, PAT_HF: High Frequency Power of PAT, PAT: Pulse Arrival Time, PH: Potential of Hydrogen, PLT: Platelets, PNN 50: Probabilistic Neural Network 50, PT: Prothrombin Time, PTT: Partial Thromboplastin Time, Paco2 = Partial Pressure of Carbon Dioxide from Arterial Blood, RR: Respiratory Rate, SBP: Systolic Blood Pressure, SD: Standard Deviation, SDPAT: Standard Deviations of Pulse Arrival time, SIRS: Systemic inflammatory response syndrome, SOFA: Sequential Organ Failure Assessment, SPo2/O2 Stat: Pulse Oximetry, SSR_HFN: The sum of squares due to regression, Temp: Temperature, UT: Urinary Tract, WBC: White Blood Cell,qSOFA: Quick Sequential Organ Failure Assessment, NR: Not Reported NI: Non Invasive

**Fig. 4:**
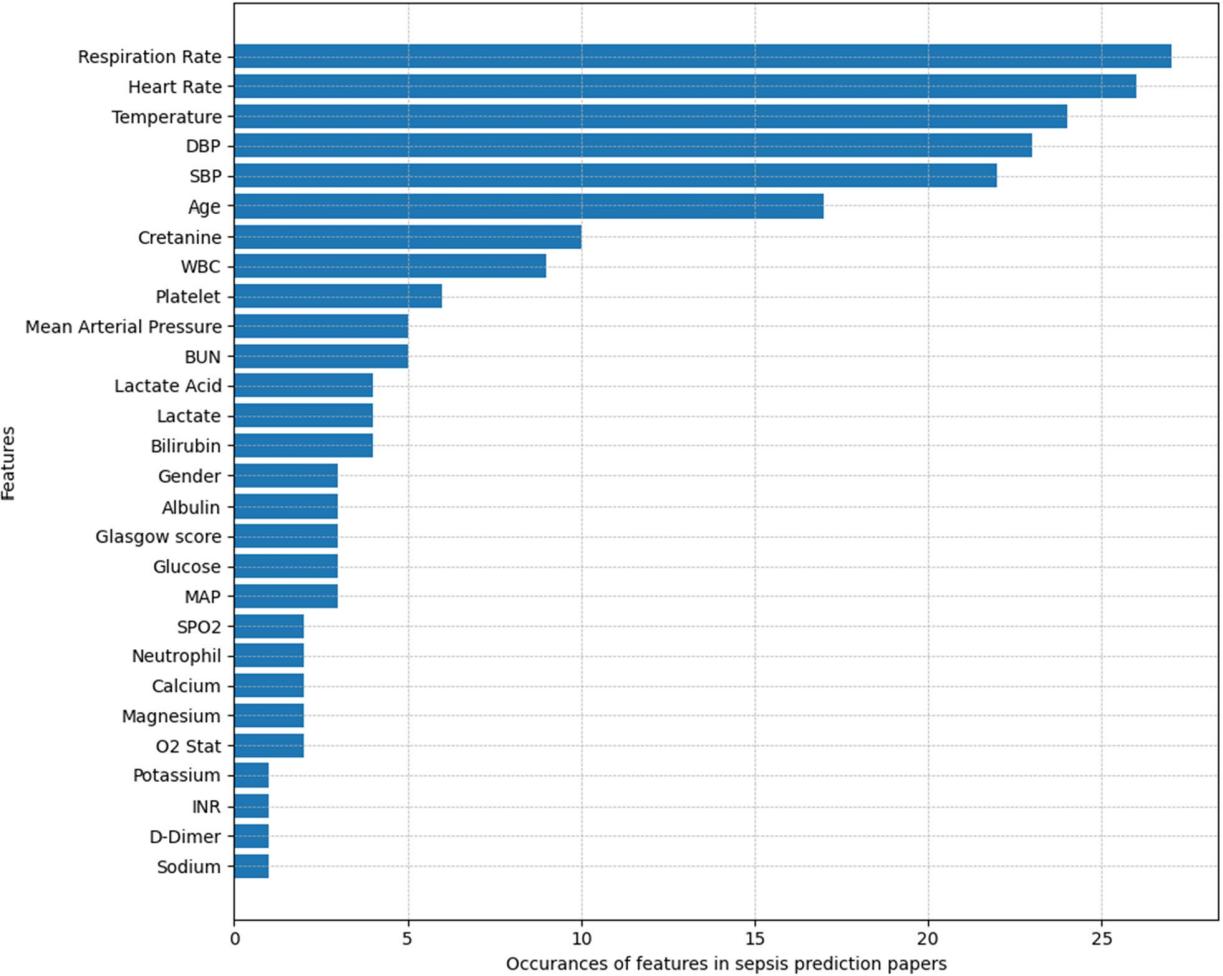
Frequency of features identified in studies

The analysis of the top 10 features, as depicted in Figure 4, highlights the most critical physiological markers for sepsis prediction. These indicators are crucial for recognizing the onset of sepsis, emphasizing the necessity of vigilant monitoring of such parameters. The graphical representation serves to underline the significant role these features play in the early detection and prediction of sepsis, pointing to the potential changes in these parameters as early signs of sepsis. This insight is vital for the development of effective early diagnosis and intervention strategies in sepsis management, illustrating the clinical importance of these markers.

To comprehensively assess the influence of various feature selection and extraction methodologies on the predictive accuracy of sepsis models, a meticulous analysis was carried out. This scrutiny was confined to investigations leveraging publicly accessible databases, namely MIMIC and PhysioNet, to ensure an unbiased comparison across diverse studies. By filtering through an expansive array of research, significant contributions from each category—Filter, Wrapper, Embedded, and Feature Extraction—were identified and their optimal results meticulously synthesized.

**Fig. 5:**
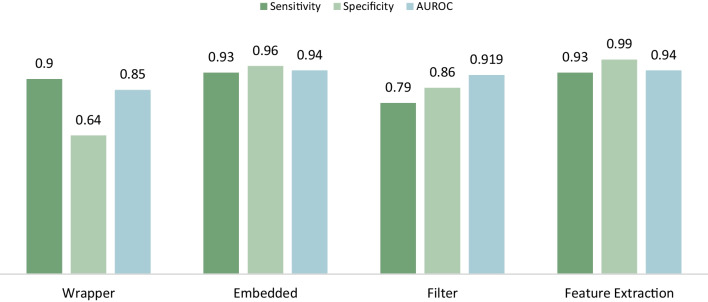
Performance analysis of different feature selection and extraction methods across open database

The graphical representation, depicted in Figure 5, elucidates the differential efficacy of these methodologies, with a particular spotlight on the Feature Extraction technique. This method emerged as notably superior, showcasing enhanced sensitivity and AUROC metrics, thereby suggesting its unparalleled effectiveness in sepsis prediction. Such findings are instrumental, indicating that feature extraction methods when specifically adapted for the nuances of sepsis prediction, are capable of significantly elevating the predictive precision of models. This detailed comparative analysis not only highlights the distinct advantages of tailored feature extraction techniques but also serves as a critical resource, offering insights into the optimization of sepsis prediction models through strategic feature selection and extraction.

#### Model performance

Table 3 synthesizes outcomes from a spectrum of studies dedicated to sepsis prediction, encapsulating the application of Machine Learning (ML) and Deep Learning (DL) strategies. It meticulously outlines the top-performing models, highlighting their Area Under the Receiver Operating Characteristic Curve (AUROC) values, Sensitivity, Specificity, and the Distribution of data for training, testing, and validation phases. The compilation reveals a broad array of algorithmic approaches, underscoring the dynamic potential of different ML models in accurately predicting sepsis. For instance, Kim et al.'s [30] bespoke model showcases exemplary performance metrics, whereas Yang et al.'s [13] study presents a contrasting scenario with their Random Forest model. This diversity in model efficacy and algorithmic application illustrates the ongoing evolution and complexity in the quest for improved sepsis prediction methodologies, aiming to significantly uplift patient care standards through enhanced diagnostic accuracy.

**Table 3:** Evaluation Measures for ML Models in Different Studies

References	ML models/deep learning	Best models	AUROC	Sensitivity	Specificity	Model split training/test/validation
Xin Zhao et al. [13]	XG Boost, Light GBM algo	GBM algo	0.98	NR	NR	75%,25%,
Rishikesan Kamaleswaran et al. [25]	XGB, LR, SVM, RF	XG boost	0.97	0.94	0.9	NR
Yash Veer Singh et al. [31]	RF, SVM, NB, LR, XG boost, Ensemble Model, Proposed Ensemble Model	Proposed ensemble model	0.96	NR	0.97	80%,20%
Supreeth P. Shashikumar et al. [27]	COMPOSER module	COMPOSER module	ICU:0.95 ED:0.95	ICU:91.6 ED:95.6	ICU:93.0 ED:93.5	80%,20%
Debdipto Mishra et al. [14])	RF, XG Boost, C5.0, Decision Tree, Boosted LR, SVM, LR, Regularized LR, Bayes General Linear Model	Random forest	0.95	83.9	0.88	80%,20%
Jae Kwan Kim et al. [32]	SOFA, qSOFA, SAPSII, LSTM, Proposed Model	Proposed model	0.94	0.93	0.91	NR
Ekanath Srihari Rangan et al. [17]	XG Boost	XG Boost	0.94	0.85	0.9	80%,20%
Kim Huat Goh et al. [17]	NLP, LDA, GBT, SERA Algo	SERA Algo	0.94	0.87	0.87	NR
Maximiliano Mollura et al. [12]	LR, XGB, KNN, MLP, SVM, TREE	LR	0.92	0.68	0.96	80%,20%,
Yongrui Duan et al. [28]	Hybrid Deep Learning Model, CNN, GRU, GBDT, DFN, DFSP	DFSP	0.92 (6)	0.8	0.87	NR
Jevier Enrique Camacho—Cogollo et al. [22]	XG Boost Model, SVM, ANN, KNN NVC, RF, Adaboost	XG Boost	0.92	NR	NR	75%,25%
Jacob Calvert et al. [28]	MLD	MLD	0.92	0.8	0.86	80%,20%
Dong Wang et al. [5]	Random forest	Random forest	0.91	0.87	0.89	80%,20%
Yu Bai et al. [18]	AdaBoost, NB, LR, Gradient, Boosted Tree, RF	AdaBoost	0.9	78.11	78.74	70%,30%
Kuo- Ching Yuan et al. [30]	XGB, DT, LR, Convolutional neural network, SVM	XG Boost	0.89	93.47	0.16	80%,20%
Alireza Rafiei et al. [4]	RNN, CNN, LSTM, SSP-LSTM	SSP-LSTM	0.89	0.74	0.74	90%,10%,NR
Heather M. Giannini et al. [35]	Random Forest, Early Warning System	Random Forest	0.88	0.26	0.98	NR
Massimiliano Greco et al. [29]	Dummy_strat, Dummy_strat*, LR, LR*, LR_balanced, LR_balanced*, RF, RF*, SOFA, APACHEII, qSOFA	Random Forest	0.86	NR	NR	90%,NR,10%
Pei Chen Lin et al. [26]	XG Boost	XG boost	0.86	IV:0.80,EV:0.67	IV:0.78 EV:0.70	80%,NR,20%
Simon Meyer Lauritsen et al. [34]	GB, Multilayer Perception, CNN-LSTM, SERA IP	CNN-LSTM	0.86 [3]	NR	NR	80%,10%,10%
Brandon DeShon et al. [1]	DeepSurv, Cox/Lasso and Cox Model	Deepsurv	0.85	0.83	0.7	70%,30%
Meicheng Yang et al. [3]	EASP	EASP	0.85	0.9	0.64	85%,15%,
Gabriel Wardi et al. [16]	AI Sepsis Expert Algo	AI Sepsis Expert Algo	85	0.85	0.68	80%,20%
Matthieu Scherpf et al. [36]	RNN, Insight algo	RNN	0.81	0.9	0.81	9/16,1/16,3/16
Donghun Yang et al. [15]	LR, Random Forest, 3 Deep Learning ANN, CNN, RNN	Random Forest	0.75	NR	NR	NR
Margherita Rosnati et al. [24]	RETAIN Model, AttTCN Model, LR, Insight model, MGP-AttTCN	AttTCN Model	0.64	NR	NR	NR
Zhengling He et al. [19]	LSTM, XG Boost, GBDT	Ensemble Model	0.40(NUS)	0.64	0.84	90%,10%
Everton Osnei Cesario et al. [20]	LSTM, Random Forest	LSTM, Random forest	AUROC-NR 0.97(ACC)	0.61	0.99	70%,10%,20%
Bilal Yaseen Al-Mualemi et al. [23]	RNN-LSTM, SVM & Adoptive CNN	Adoptive CNN	0.78	0.93	0.93	NR

APACHE II: Acute physiology and chronic health evaluation II, AUROC: Area Under the Receiver Operating Curve, Att-TCN: Attention Temporal Convolutional Network,CNN: Convolutional Neural Network Dummy_strat: Dummy stratifier (baseline comparison), DFN: Deep Functional Network, DFSP: Double Fusion Sepsis Predictor, EASP: Explainable AI Sepsis Predictor Model, EV: External Validation, GB: Gradient Boosting, GBDT: Gradient Boosting Decision Tree, GRU: Gated Recurrent Unit, IV: Internal Validation, KNN: K-Nearest Neighbours,LDA: Latent Dirichlet allocation, LR: Logistic Regression, LSTM: Long Short Term Memory, MLD: Machine Learning Based Diagnostic, MLP: Multilayer Perceptron, NB: Naïve Bayes NUS: Normalized Utility Score, RF: Random Forest, RNN: Recurrent Neutral Network, SERA: Sepsis Early Risk Assessment, SOFA: Sequential Organ Failure Assessment, SSP: Smart Sepsis Predictor, SVM: Support Vector Machine, XGB: Extreme Gradient Boosting, qSOFA: quick Sequential Organ Failure Assessment, NR: Not Reported

### Impact of prediction time window on model performance

**Fig. 6:**
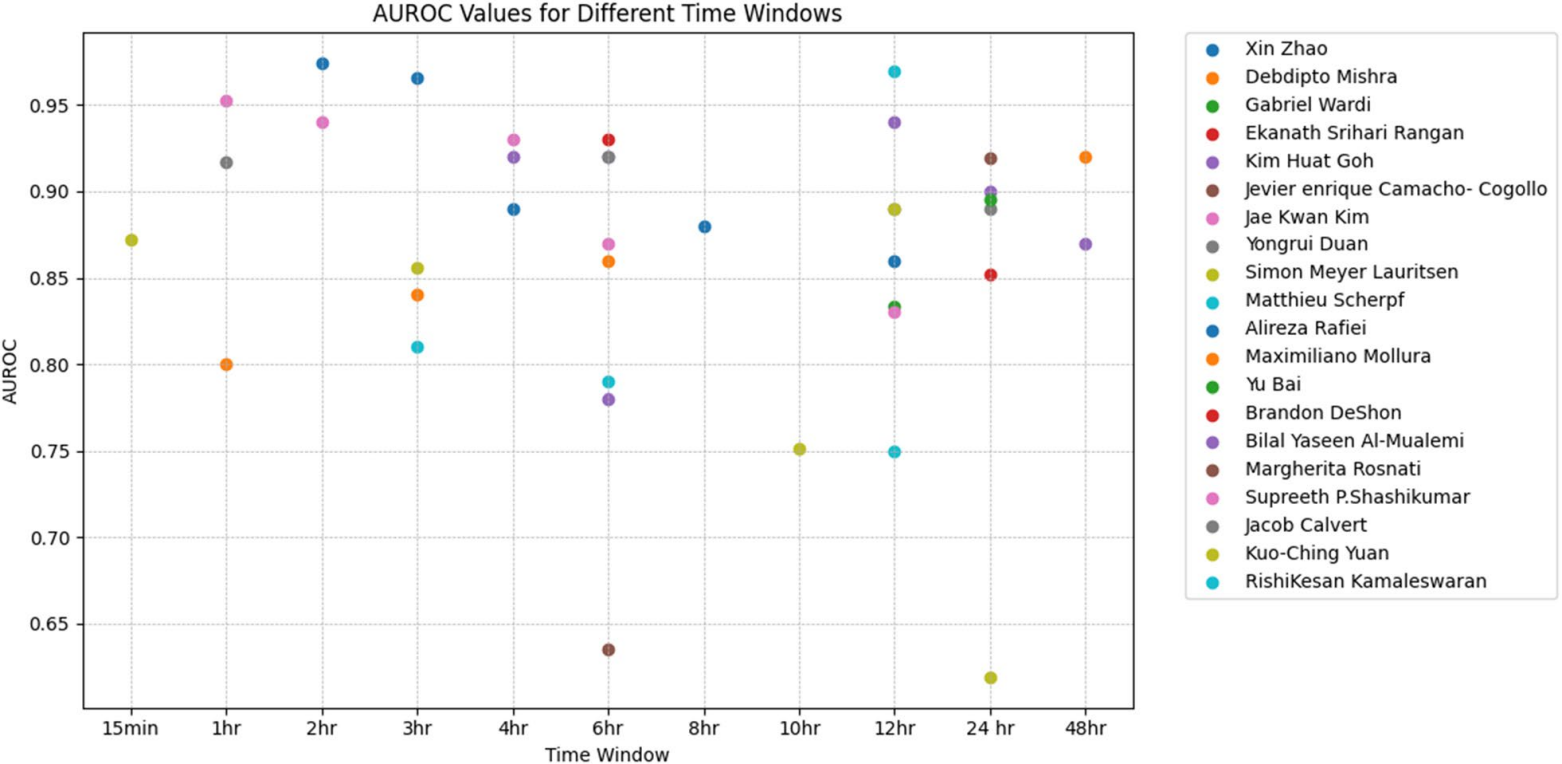
Predicting the performance of multi-time window

The prediction time window is crucial as it plays an important role in clinical intervention, resource allocation, treatment planning, false positive rates, clinical workflow, and model evaluation. Figure 6 depicts the impact of the different prediction time windows on the model performance.

Some studies including [18, 21, 34] showed that the ML model gave dependable results (higher AUROC while minimizing false positive and false negative rates) when predicting sepsis at different time intervals 12, 24 and 48 h. These results have clinical significance as they demonstrate that the model’s predictive power remains consistent across the crucial time windows. It’s also worth noting that some studies [12–14, 32–34, 36] have shown consistent results across early time points (1 to 6 h) which indicates that they can identify septic cases in their early stages.

In the course of this investigation in a study [4], a novel Smart Sepsis Predictor (SSP) model was meticulously developed, employing a Recurrent Neural Network (RNN) architecture. The SSP model was thoughtfully designed to operate in two distinct modes, each harnessing crucial inputs encompassing a spectrum of patient data, including vital signs, demographics, and laboratory values. What sets this model apart is its remarkable proficiency in achieving higher Area Under the Curve (AUC) scores when applied to a 12-h prediction window. This capability arises from its unique capacity to discern intricate and nuanced relationships within vital sign data, thereby facilitating timely alerts to healthcare practitioners. It is noteworthy that the findings reported herein align consistently with the outcomes observed in prior studies, specifically references [34] and [36].

This study [31] introduced a novel early warning model known as the Double Fusion Sepsis Predictor (DFSP), which stands as a hybrid deep-learning framework amalgamating deep features with meticulously engineered attributes encompassing statistical metrics and clinical scores. The outcomes of this investigation present compelling evidence for the superior performance of DFSP when juxtaposed with a pure deep learning model. Specifically, DFSP demonstrates a substantial enhancement in the Area Under the Receiver Operating Characteristic (AUROC) curve across 6, 12, and 24-h prediction horizons. This improvement is attributed to the utilization of fusion strategies, which not only enhance predictive capabilities but also significantly elevate the AUROC scores.

In this study [19], an advanced Sepsis Early Risk Assessment (SERA) algorithm was devised, incorporating both structured and unstructured clinical notes. Through data mining techniques, the SERA algorithm demonstrated enhanced predictive accuracy compared to utilizing solely clinical metrics. The Receiver Operating Characteristic (ROC) analysis of the SERA algorithm consistently surpassed predictions made by physicians across all examined time intervals, exhibiting notably high Area Under the ROC Curve (AUROC) scores even up to 48, 24, 12, 6, and 4 h preceding the onset of sepsis.

## Discussion

From the list of 29 included studies, almost all of them, ICU-based studies (68%) and ED-based studies (13%), were conducted in critical care settings in the hospitals, thus showing the significance of ML in critical care data analytics. Early diagnosis and treatment play an important role in reducing the mortality due to sepsis, but advanced and accurate detection of sepsis is still a challenge in the clinical domain. When we discuss the electronic monitoring of sepsis patients for predicting and detecting early symptoms of complications, that's where the ML algorithms come in and play their role by identifying patterns and relationships from vast / big patients' datasets to solve this complex problem [37]. While reviewing the related literature, we found several studies using ML models and algorithms for sepsis prediction as mentioned in the above results section. Linked with the subject of our current study, we found three important scoping reviews / meta-analysis that focused on the potentials of ML for sepsis prediction [2, 38,39]. The review from Deng et al. included 21 studies focusing on early sepsis detection, prediction and mortality. It concluded that no model could be adopted widely yet in general due to the lack of unified validation standards / procedures and the heterogeneity in patients’ cohort, though it referred Deep Neural Networks (DNNs) as more suitable tool as compared to the other traditional tools for high-dimensional and highly heterogeneous patients' sepsis data. Interestingly, it recommended using ML as a feature engineering tool, which reflects the need for and importance of our conducted study in this field; and suggested AUROC as evaluation standard for model performance as in our results. The review and meta-analysis from Fleuren et al. [38] showed ML models prediction for Sepsis ahead of time using retrospective data by examining 28 included studies out of which 24 reported AUROC as their performance metric in critical care settings. and focused on AUROC to analyze model performance whereas we looked at the other metrics like sensitivity and specificity in addition to AUROC. Though the results of this review showed that individual models outperformed the traditional scoring tools, the authors suggested the need of development of reporting guidelines for ML models in critical/intensive care settings and their implementation with diverse patient populations to see the clinical impact. Similarly, another meta-analysis study from Islam et al. [39] included seven observational studies to quantify the performance of ML models for Sepsis prediction. The outcomes showed that.

ML prediction models performed well as compared to existing sepsis scoring systems, such as SIRS, MEWS, SOFA, and qSOFA for identification and prediction of sepsis patients; and suggested for more multi-centered studies with more precise clinical variables for sepsis prediction in the future. In contrast to these review / meta-analysis studies, our review dedicatedly focused on different critical features and feature extraction methods. The results of our study showed the key dynamic features that are pivotal in early sepsis prediction; demonstrated the critical role of feature selection methods in enhancing the efficacy of predictive models in sepsis; and proved the effectiveness of feature extraction models—Random Forest and XG Boost with high sensitivity and AUROC in facilitating the sepsis prediction, and DL showing excellent AUROC values for different predicting time windows (12–48 h.). Concisely, the increased accuracy of sepsis prediction using these ML models can lead to minimizing the hospital mortality rate, reducing the LOS, improving the patient safety, and at the same time saving millions of dollars of investment in large clinical settings, hence proving the potentials and importance of these models in this domain.

This scoping review has several strengths. It followed a comprehensive and systematic approach to assess the landscape of sepsis prediction using ML techniques. It offered a thorough compilation of feature selection and extraction techniques used in the sepsis prediction and identified the top features for sepsis prediction across all studies. Additionally, by categorizing the studies based on features, prediction time and model performance, this study provided a clear comparison of different approaches that can support researchers and healthcare professionals in informed decision making.

There were certain limitations related to features. Firstly, the feature variability, the studies examined in this review utilize a wide array of features reflecting the diversity of clinical data sources and methodologies. However, the variability in selected features across studies can hinder direct comparisons and the identification of universally impactful features. Secondly, the features that prove influential in one clinical context may not necessarily generalize to other healthcare settings or patient populations. Thirdly, many studies identified critical features, but not all provided in-depth insights into the clinical significance or mechanistic explanations of these features. Lastly, this review was constrained by the availability of data in the studies analyzed, as incomplete or restricted datasets can lead to incomplete representation of potentially critical features.

We hope that this review will help clarify which features and methods are most promising for improving the accuracy of sepsis prediction models for future research studies. Ultimately, the findings from this review will be valuable not only for researchers but also for healthcare professionals, as they seek to enhance early sepsis detection and patient care.

## Conclusion

To our best knowledge, this is the first study of its kind that reviewed critical features and feature extraction methods for sepsis prediction. Spanning diverse studies, it encompassed over 18,841 features and explored techniques like wrapper, filter, and embedded extraction to assess their impact on sepsis prediction models. The findings of this study highlighted the pivotal role of dynamic features, notably encompassing vital signs, such as Temperature, Heart Rate, and Blood Pressure, alongside critical laboratory parameters including White Blood Cell count (WBC), Creatinine, Bilirubin, Platelet count, and Lactate levels in sepsis prediction. These dynamic features have shown consistent and substantial prominence in prognosticating the onset of sepsis, exhibiting remarkable discriminatory power and pivotal utility in the early detection of septic conditions. In contrast, the demographic variables have evinced comparatively diminished influence in effectively predicting sepsis. For enhancing the predictive efficacy of sepsis models, the strategic implementation of feature selection methodologies has emerged as a crucial factor. The judicious identification and integration of key predictors via Filter, Wrapper, and Feature extraction techniques, these methodologies have effectively mitigated data dimensionality issues and conferred enhanced model stability, thereby facilitating the development of accurate and refined predictive models. In terms of model efficacy, the Random Forest and XG Boost models have exhibited superior performance, with commendable AUROC, sensitivity, and specificity scores. Additionally, Deep Learning models have demonstrated consistent and profound insights into the correlation between features and model predictions, an aspect that conventional Machine Learning models have not been able to fully elucidate yet. These Deep Learning models have demonstrated remarkable AUROC values across different prediction time windows, ranging from 12 to 48 h.

We recommend standardization of feature engineering methods used in sepsis prediction models which will facilitate comparison across studies and will foster consistency. Also, researchers should provide detailed description of the feature engineering process in their publication, including the rationale behind selecting methods and detailed data preprocessing steps.

In summary, this study reaffirmed the crucial role of features in sepsis prediction. The careful choice of feature extraction methods can significantly impact the model’s performance and provide clinicians with valuable insights into complex interrelationships.

## Data Availability

Data supporting the findings of this study are available from the corresponding author, upon reasonable request.
